# Mr. Bellot's Case of Strictured Intestine

**Published:** 1809-11

**Authors:** Abraham Bellot

**Affiliations:** Oldham, Lancashire


### *To the Editors of the Medical and Physical Journal.*

GENTLEMEN,

MRS. J. L. ætat. 26, in October 1805, was seized with violent inflammation and obstruction in the bowels, which was removed by the usual means employed in similar cases; she was naturally of a very costive habit, and after this severe

---

severe attack, her bowels became still more irregular, being frequently several days without having any evacuation, or voiding only sybala; then generally came on a diarrhœa, which often alternated with the obstipation. She continued several months in this irregular way; sometimes being much troubled with piles, and almost constantly complaining of some uneasiness, with a bearing down about the seat. She now began to void a kind of slimy matter per anum; this discharge continued several months, but at length gradually became more purulent in its appearance; different kinds of aperient medicines were ordered to regulate the bowels, and also occasional anodynes, with bitters and tonics; these afforded temporary relief. In the month of January 1807, she became pregnant; this unfortunately added to her sufferings, as it tended to increase the costiveness, and also deterred her from taking her usual aperients: every attention was paid to her diet, but this had very little effect. As she advanced in pregnancy, her pains kept increasing, and her situation still more distressing, for the obstruction which was suspected to arise from stricture, resisted, in a great measure, all medicines; glysters were daily administered, which afforded some relief, by procuring small evacuations; the glysters had frequently to be repeated, as they often returned without bringing away any fæces. When she was so extremely costive, it became necessary to give repeated doses of aperient medicines, which were taken sometimes two or three days together, and then their operation was often rather violent. It was proposed to try what bougies would do in this case, towards removing, or at least preventing the stricture getting worse: the situation of which, had been previously ascertained to be about the length of the finger from the verge of the anus. Mrs. L——'s pains were so great at this time, that premature labour was daily expected to take place. A situation so precarious, prevented the regular use of bougies. Soft bougies were two or three times introduced, which, having passed above the length of the finger, occasioned some little pain; the stricture at this time appeared not to be very firm, as very little impression was made upon the bougie; at length her situation and aversion to them, obliged us to give them up till after delivery, and for the present to confine ourselves to a palliative mode of treatment.

In the month of August she was delivered, when about seven months advanced in pregnancy; the child died the same evening. The delivery was speedy, and without any extraordinary extraordinary pain; she appeared to be quite as well as could be expected. Some days after, a violent aphthous *fever* came on, attended with a severe diarrhœa, and violent pains in the bowels; the aphthous affection of the mouth and throat continued very bad, and seemed to extend to the stomach and bowels, from the heat and pain which were felt in swallowing any thing a little warm, or the least irritating. Demulcents combined with astringents and opiates were given freely, which afforded considerable relief, though the complaint continued very obstinate for a length of time, and brought our patient very low and weak. The bougies, which we intended trying again, she was extremely averse to, and from the bowels continuing so very soluble, at the same time she was suffering so much pain, that their use was not thought absolutely necessary; indeed, some hopes were now entertained, that the stricture was better, as no purulent matter had appeared whilst the bowels remained so very soluble; but all our hopes were entirely delusive, when the diarrhœa was moderated, the purulent matter was again voided, even in greater quantity, which showed there must be some extensive ulceration. There might be some matter voided during the diarrhœa, but being so intimately mixed with the loose fæces, could not readily be perceived. Her sufferings now become great beyond description, and could only be temporarily alleviated by large and repeated doses of opium; her appetite decreased, with a general wasting, and all curative intentions were now given up, as there appeared no hopes whatever of recovery. Her situation became now the most distressing; the ulceration appeared to have extended so far, as to form an opening of communication between the intestine and bladder, or the vagina, through which wind passed, and also feculent matter, which was voided along with the urine; to add still more to her sufferings, a violent inflammation took place in the right groin; this was supposed to arise from some matter burrowing in the cellular membrane; anodyne poultices were applied to mitigate the pain; but happily, in a short time, the most powerful and effectual anodyne was administered, by death putting an end to a most painful and miserable existence.

Leave being obtained to open the body, the diseased parts were attentively examined by Dr. Hull and myself, and the following were the appearances: The right groin, which had become inflamed, was very black, and hastening fast to putrefaction; the labia pudendi were much inflamed, and there there was some ulceration about the anus externally. On opening the cavity of the abdomen, in cutting through the skin and muscles down to the right ileum, a large quantity of feculent matter was found in the cellular membrane; which, by an ulcerative process, must ere long, have made its escape by an opening in the groin. An adhesion had taken place here, between the cæcum and peritoneum; ulceration afterwards followed, which destroyed the coats of the intestine, and through this opening the feculent matter was making its way externally; considerable adhesions were likewise discovered between the bladder and ileum, where this portion of the intestine terminates in the cæcum, and also between the ileum and sigmoid flexure of the colon. For a more accurate examination, the intestines, uterus, and bladder were taken out; the intestines were so much diseased, that in drawing them out to separate them completely, they were lacerated; a large quantity of fæces was accumulated in the large intestines. When we had removed the whole contents, and cleared away the fæces, an examination was begun from the rectum, which was cut off close to the anus, with as much of the vagina as possible, and also the bladder; in the rectum, exactly the length of the forefinger from the anus, commenced a stricture, which was about four fingers in breadth, and about the middle was the narrowest part; here it would scarcely admit the little finger; just above the stricture, the intestine formed a sort of pouch, where the fæces had lodged, not being able to pass the strictured part till their diameter was lessened. On the back part of this pouch, an opening was formed, which would nearly admit two fingers; this led to a large cavity or sinus behind the rectum, between it and the os sacrum and coccygis, in which was contained a great quantity of feculent matter; and if the patient had lived a little longer, this would have made its way by a fistulous opening near the anus. There was a considerable dilatation of the rectum as far as the sigmoid flexure of the colon, where there was an adhesion of this part of the intestine and the ileum; through the parts where they were adhering, ulceration had taken place, so as to form a communication between the large and small intestines; by which opening, chyme, if it may be so called, at this part of the ileum, had sometimes passed from the ileum into the sigmoid flexure of the colon, and voided in this state per anum; this was partly proved by the circumstance stance of the patient having voided by stools small lumps of suet, at the time she was taking it mixed with milk. A regurgitation of fæces would likewise frequently take place, from the rectum into the ileum, through the same opening, when there was any quantity accumulated in the pouch above the stricture. Part of the fæces then must often pass the same round through the large intestines, twice, or perhaps oftener, before it could be voided per anum. A little further from this opening were two small apertures leading into the bladder, to which part the termination of the ileum and beginning of the cæcum were adhering at its fundus; through these small apertures must have passed the feculent matter, which had been regurgitated from the large into the small intestines, and from thence into the bladder. It was now easily accounted why the urine was in general intimately tinged with feculent matter when voided, but afterwards separated by standing. Some very small bits of fæces were sometimes seen in the urine. It is wonderful to remark, in this case, the care which Nature took in guarding the internal cavities; for though such extensive ulceration had taken place, no fæces or matter had escaped into the abdominal cavity; had this taken place, violent peritoneal inflammation would have come on, which must in a short time have terminated in the death of the patient; but, instead of this, after the stricture had become permanent, nature was attempting to relieve herself three different ways; and there is every reason to believe, all the apertures would have been external; viz. the small apertures into the bladder, which had already taken place; the next in the groin, which would have been in a few days; and the third, near the anus, which, ere long, would have taken place, had the patient lived. Yours, &c.

ABRAHAM BELLOT.

*Oldham, Lancashire,*
*Oct.* 2, 1802.

